# Three Single Nucleotide Polymorphisms of *LOXL1*’ in a Turkish Population with Pseudoexfoliation Syndrome and Pseudoexfoliation Glaucoma

**DOI:** 10.4274/tjo.83797

**Published:** 2018-10-31

**Authors:** Yetkin Yaz, Nilgün Yıldırım, Yasemin Aydın Yaz, Oğuz Çilingir, Zafer Yüksel, Fezan Mutlu

**Affiliations:** 1 Eskişehir Yunus Emre State Hospital, Ophthalmology Clinic, Eskişehir, Turkey; 2 Eskişehir Osmangazi University Faculty of Medicine, Department of Ophthalmology, Eskişehir, Turkey; 3 Eskişehir State Hospital, Ophthalmology Clinic, Eskişehir, Turkey; 4 Eskişehir Osmangazi University Faculty of Medicine, Department of Medical Genetics, Eskişehir, Turkey; 5 Eskisehir Osmangazi University Faculty of Medicine, Department of Biostatistics, Eskişehir, Turkey

**Keywords:** Pseudoexfoliation syndrome, pseudoexfoliation glaucoma, LOXL1 gene

## Abstract

**Objectives:**

To investigate the three single nucleotide polymorphisms (SNPs) (rs3825942, rs1048661, and rs2165241) of the *LOXL1* gene in pseudoexfoliation syndrome (XFS) and pseudoexfoliation glucoma (XFG) in the Turkish population.

**Materials and Methods:**

DNA was obtained from blood samples of 48 XFS, 58 XFG, and 171 control subjects. Three *LOXL1* SNPs (rs3825942, rs1048661, rs2165241) were investigated with real time PCR, a probe-based genotyping method, and melting curve analysis.

**Results:**

All three SNPs of *LOXL1* were significantly associated with XFS (rs3825942 p=3.54x10^-6^, odds ratio [OR]=∞; rs1048661 p=0.008, OR=2.18; rs2165241 p=8.69x10^-9^, OR=4.30) and XFG (rs3825942 p=3.41x10^-7^, OR=∞; rs1048661 p=1.75x10^-5^, OR=3.78; rs2165241 p=3.85x10^-11^ OR=4.90). No significant differences were observed between the XFS and XFG groups for any of the SNPs. The GG genotype of rs3825942 was more valuable for distinguishing pseudoexfoliative cases from healthy individuals. The homozygous TT genotype of rs2165241 was associated with 6-fold increased XFS risk (p=8.15x10^-8^, OR=6.32) and 7-fold increased XFG risk (p=1.45x10^-10^ OR=7.95). The GGT haplotype consisting of all three risk alleles was associated with a 7.45-fold higher risk of XFS/XFG (p=8.65x10^-14^, OR=7.45). Presence of T allele of rs2165241 conferred 3 times higher risk for men than women (p=6.78x10^-5^, OR=3.202).

**Conclusion:**

*LOXL1* SNPs are associated with increased risk for pseudoexfoliation in the Turkish population. T allele of rs2165241 was found to be the most important characterized risk factor for our cohort. All SNP distributions were similar to other European and American populations.

## Introduction

Pseudoexfoliation glaucoma (XFG) is the most common identifiable cause of secondary open-angle glaucoma worldwide.^1^ Pseudoexfoliation is an age-related systemic disorder characterized by progressive accumulation of abnormal fibrillar extracellular material in ocular and extraocular tissues.^1,2^ Pseudoexfoliation syndrome (XFS) is diagnosed based on the appearance of pseudoexfoliation material (PXM) on anterior segment structures like the pupillary border of the iris or the anterior lens capsule. XFS has been associated with increased risk of cataract and glaucoma^3,4^ and predisposition to a broad range of intraocular complications during surgery.^1,2^

The structure and origin of PXM has not been determined. A recent genome-wide association (GWA) study identified the *LOXL1 *gene, which encodes an enzyme necessary for elastogenesis and collagen cross-linking, as a major genetic risk factor for developing PXF.^5,6^ Three single nucleotide polymorphisms (SNPs) have been associated with PXF: one intronic SNP, rs2165241 located in intron 1, and two nonsynonymous coding SNPs, rs3825942 (G153D) and rs1048661 (R141L) located in exon 1.^5^ However, the frequency of risk alleles for both variants varies among different populations. Based on these studies, *LOXL1* is necessary but not sufficient for PXM development.^5^ Due to the relationship between PXM, the extracellular matrix, and basement membrane, the role of different genes were investigated in PXM formation. These genes were related to elastic fiber and extracellular matrix metabolism, such as MMP1, MMP3, FBN1, LTBP2, MFAP2, TGM2, TGF-b1, and CLU.^7,8,9^ In addition, *LOXL1 *promoter haplotypes, which influence gene expression and lead to reduced enzyme activity, are associated with XFS/XFG.^5^

The purpose of this study was to investigate variant SNPs (rs2165241, rs3825942 and rs1048661) of the *LOXL1* gene to determine the association of *LOXL1 *polymorphism with XFG and XFS in the Turkish population.

## Materials and Methods

### Study Population

The study population comprised 277 individuals including 58 patients with XFG, 48 patients with XFS, and 171 healthy age- and sex-matched controls. Written informed consent was obtained from all participants. The study was approved by the ethics review board of the Eskişehir Osmangazi University Faculty of Medicine and adhered to the tenets of the Declaration of Helsinki.

All participants were questioned about systemic diseases (diabetes, hypertension, thyroid, and rheumatic diseases) and drug usage, then underwent a standardized detailed ophthalmic examination, which included refraction, visual acuity, intraocular pressure (IOP) (Goldmann applanation tonometry), anterior segment biomicroscopy examination, and fundus examination. XFG was defined as the presence of PXM on the anterior lens capsule or pupillary margin, elevated IOP (≥21 mmHg), glaucomatous optic disc changes (vertical cup-to-disc ratio [C/D] ≥0.5, C/D asymmetry ≥0.2), characterized visual field defects in computed perimetry (Zeiss Humphrey visual field analyzer white-to-white 30-2 threshold program). Patients with PXM on the anterior lens capsule and pupillary margin but whose IOP, optic disc, and visual field findings were within normal limits were defined as XFS. Individuals without clinical signs of XFS/XFG were recruited as a control group. Demographic and clinical characteristics of the groups are presented in Table 1.

### DNA Extraction and Genotyping

Venous blood samples (5 mL) were collected from the antecubital region to investigate *LOXL1* gene polymorphism. The samples were collected in NaEDTA tubes and stored at -20 ˚C.

Roche Magna Pure Compact robotic DNA isolation system protocol was used for DNA extraction. Blood samples were put directly into the robotic DNA isolation system. Sample volume 200 µL, elution volume 100 µL and DNA isolation blood protocol were selected. The robotic system consists of proteinase K, irrigation solution, magnetic particles for DNA isolation, cartridge system for pipetting, tip trailer for pipette tips, and rack for sample and elution tubes. All of the steps were performed automatically except arranging the cartridge and tip trailer, and putting the samples and elution tubes into the robotic system. The procedure lasted approximately 25 minutes for each set of 8 samples. DNA samples were stored at -20 °C.

Amplification of isolated DNA samples with real-time PCR and melting analysis: After the replication of the SNPs to be investigated by Roche LightCycler 480 Real Time PCR, real-time PCR melting curve analysis was performed by using hybrid probe kits designed specifically for SNPs. Mutant types were determined with melting curve analysis by evaluating differences in melting temperature degrees of SNPs. Three hundred and eighty-four RXN Molbiol LightSNiP^®^ kits which were designed for specific SNP regions were used for melting analysis according to the manufacturers’ recommendations.

Reaction mixtures were prepared with LightCycler^®^ FastStart DNA Master HybProbe, then put into plates and DNA was added. The recommended program was used in the real-time PCR device.

Fluorescence occurred at different temperatures for each allele. For rs3825942, signals detected at 62.21 °C were evaluated as G allele and those at 69.73 °C as A allele; for rs1048661, signals at 54.42 °C were evaluated as G allele and at 65.82 °C as T allele; for rs2165241, signals detected at 52.34 °C were evaluated as C allele and at 59.30 °C as T allele.

### Statistical Analysis

The incidence was calculated as a percentage for each genotype. Allele and genotype frequencies in the patient and control groups were compared with values predicted by Hardy-Weinberg equilibrium using the chi-square test. Kruskal-Wallis test was used for comparison of age, IOP, and C/D between the groups. Dunn’s multiple comparison test was used for variables that showed differences among groups. Continuous variables were assessed using the Shapiro-Wilk test. Continuous variables were expressed as median (25%-75%) and categorical variables were shown as frequencies (percentages). Pearson and Yates correction chi-square tests were done for comparisons of allele and genotype variables between the groups. Odds ratios (OD) and 95% confidence interval (CI) were calculated. A p value of <0.05 was considered of statistical significance. The statistical analyses were performed using SPSS version 20.0 (SPSS Inc., Chicago, IL, USA).

## Results

A total of 277 individuals (58 XFG, 48 XFS, 171 controls) over 40 years old were recruited for this study in Eskişehir Osmangazi University Department of Glaucoma. The mean age was 68 (66.5-70) years for the XFS group, 69 (68-73.25) years for the XFG group, and 67 (64-70) years for control subjects. 

The genotype distribution of all SNPs conformed to Hardy-Weinberg equilibrium. In both XFS and XFG, a strong association with the risk allele of each individual SNP (rs2165241T, rs1048661G, and rs3825942G) was observed (Table 2). The G allele of rs3825942 was present in all XFS and XFG patients, thus OR could not be calculated (OR=∞ p=3.54x10^-6^, OR=∞, p=3.41x10^-7^). In the control group, G allele for rs3825942 was more common than the A allele. For rs3825942, all patients in the XFS and XFG groups had the GG genotype, and again ORs could not be calculated (OR=∞ p=1.57x10^-6^, OR=∞, p=1.45x10^-6^). The G allele and GG genotype of rs1048661 were detected more frequently in all groups. The T allele of rs1048661 was underrepresented in patients with XFS (OR=2.18 95% CI=1.21-3.91, p=0.008) and XFG (OR=3.78 95% CI=1.99-7.18, p=1.75x10^-5^) when compared to control subjects. No TT genotype of rs1048661 was detected in XFS (OR=1.93 95% CI=0.98-3.77, p=0.076) or XFG (OR=3.71 95% CI=1.83-7.47, p=2.75x10^-14^) patients. In control subjects, the TT genotype of rs1048661 was detected more frequently when compared with XFS and XFG patients. However, the GG and GT genotype of rs1048661 were overrepresented in control subjects when compared to the TT genotype of rs1048661. For rs2165241, the T allele was more frequent than C allele in XFS (OR=4.30 95% CI=2.55-7.25 p=8.69x10^-9^) and XFG (OR=4.90 95% CI=2.98-8.06 p=3.85x10^-11^) patients, while in control subjects the C allele was more common. The genotype distribution of rs2165241 was different in XFS and XFG patients and control subjects. The TT genotype of rs2165241 was overrepresented and the CC genotype of rs2165241 was underrepresented in XFS (OR=6.32 95% CI=3.16-12.64, p=8.15x10^-8^) and XFG (OR=7.95 95% CI=4.10-15.42, p=1.45x10^-10^) patients when compared to control subjects. The CT genotype was the most frequent genotype in control subjects. For three SNPs the TT genotype of rs2165241 was detected more frequently in XFS (58%) and XFG (63%) when compared to control subjects (19%). Likewise, the homozygous TT genotype of rs2165241 was associated with 6.32-fold higher risk of XFS (95% CI=3.16-12.64) and 7.95-fold higher risk of XFG (95% CI=4.10-15.42).

For three SNPs, rs3825942, rs1048661, rs2165241, haplotype analysis of risk alleles was calculated to determine the combined effects on pseudoexfoliation patients (XFS+XFG) and control subjects (Table 3). The haplotypes consisting of risk alleles were overrepresented in pseudoexfoliation patients as compared to control subjects for each SNP (p=8.65x10^-14^) and were associated with 7.45-fold increased risk of pseudoexfoliation (95% CI=4.22-12.99).

According to previous studies, females are affected by XFS more often than males, whereas XFG is more severe in males than females.^2^ In our allele analysis of all pseudoexfoliation patients (XFS+XFG) based on gender, the T allele of rs2165341 was detected in 75% of males and 49% of females (Table 4). Existence of T allele was associated with 3.2 times higher risk for men than women (p=6.78x10-5, c^2^=15.871, OR=3.202 95% CI=1.719-5.989).

## Discussion

After Thorleifsson et al.^5^ from Iceland identified three associated polymorphisms of *LOXL1* in XFS/XFG, many studies were performed on Caucasian, Asian, and African populations.^10,11,12,13,14,15,16,17,18,19,20,21,22,23,24,25,27,28,29,30,31,32^ In our study we investigated the association between three SNPs of *LOXL1 *(rs1048661, rs3825942, rs2165241) and XFS/XFG in the Turkish population. Our findings show a significant association with XFS, as in other recent studies. However, geographically Turkey is located in both Asia and Europe, and this association in our population was similar to that observed in Caucasians, as in European populations.

In our study, the relationship between G allele and GG genotype of rs3825942, G allele and GG genotype of rs1048661, and T allele and TT genotype of rs2165241 was found in XFS/XFG, as in the Caucasian population.^10,11,12,13,14,15,16,17,18,19,20,21,22^ In the present study the G allele and GG genotype of rs3825942 was present in all XFS and XFG cases. According to the results of an African study and another Turkish study^11^ for rs3825942, the A allele carries an increased risk for XFS.^11,32^ However, in our study and another study from Turkey, the A allele was not detected in any XFS and XFG cases and was suggested to be protective.^10^ Similarly, in another Turkish study, the G allele GG genotype of rs1048661 were detected in exon 1 of *LOXL1*, as seen in the patients in our study group.^12^

Studies in European and American populations reported that G allele and GG genotype of rs1048661 and T allele and TT genotype of rs2165241 were associated with XFS, whereas studies in Asian populations identified the opposite relationship for risk alleles and genotypes for these SNPs.^23,24,25,27,28,29,30,31^ Our results appear to be similar to Caucasians but different from Asians in terms of allelic and genotypic distributions of rs1048661 and rs2165241.^10,11,12,13,14,15,16,17,18,19,20,21,22^ Another study from Turkey indicated that T allele and TT genotype of rs2165241 were associated with XFS/XFG, like our study, but revealed no significant relationship with G allele and GG genotype of rs1048661.^13^ For this reason, the pathogenesis of XFS cannot be explained by genetic factors alone.

In several studies researching whether *LOXL1 *gene polymorphism has any role in predicting XFG development, no significant association was found in the differentiation of XFS and XFG.^1^ Likewise, in our study, no differences in *LOXL1 *polymorphism were found between the XFS and XFG groups. On the other hand, recent studies have shown that females were affected more frequently by XFS than males, whereas XFG was more severe in males compared to females.^2^ We observed a strong relationship between the T risk allele in the rs216341 SNP and gender in XFS and XFG, with men carrying the T allele showing at 3 times higher risk of disease. However, a study from Japan did not show significant gender differences in any of the three SNPs.^28^

In our study, the haplotype (GGT) consisting of all three risk alleles of *LOXL1 *SNPs (rs3825942 G, rs1048661 G and rs2165341 T) was associated with a 7.45-fold higher risk of XFS/XFG. A study in an American population reported a 3-fold higher risk^6^ and a study in a Polish population determined a 4-fold higher risk with the GGT haplotype.^22^

## Conclusion

Our findings are similar to previous Turkish studies that investigated two non-synonymous coding SNPs rs3825942 and rs1048661, but we observed intronic SNP rs2165341 as well. Our results support the existence of a significant association between three SNPs of LOXL1 with both XFS and XFG, though no significant differences were found between the XFS and XFG patients. Also different from the genotypes of exonic SNPs (rs3825942, rs1048661), the TT genotype of rs2165341 was detected more frequently in pseudoexfoliation and was associated with 6-fold and 7-fold increases in risk of XFS and XFG, respectively. Likewise, we observed a relationship between the T allele of rs2165341 and gender, as presence of the T allele was associated with 3 times higher risk for men compared to women. To the best of our knowledge, rs2165341 is an important and risk-modifying factor in our cohort. In conclusion, LOXL1 gene polymorphism has a significant influence on XFS and XFG pathogenesis, but is inadequate to explain the exact mechanism. Therefore, further genetic and epigenetic studies are needed.

## Figures and Tables

**Table 1:**
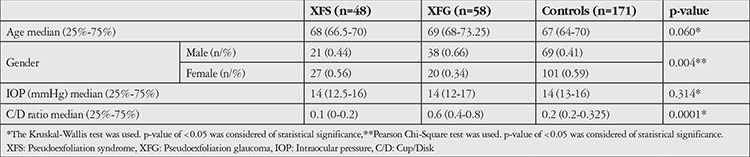
Demographic and clinical characteristics

**Table 2:**
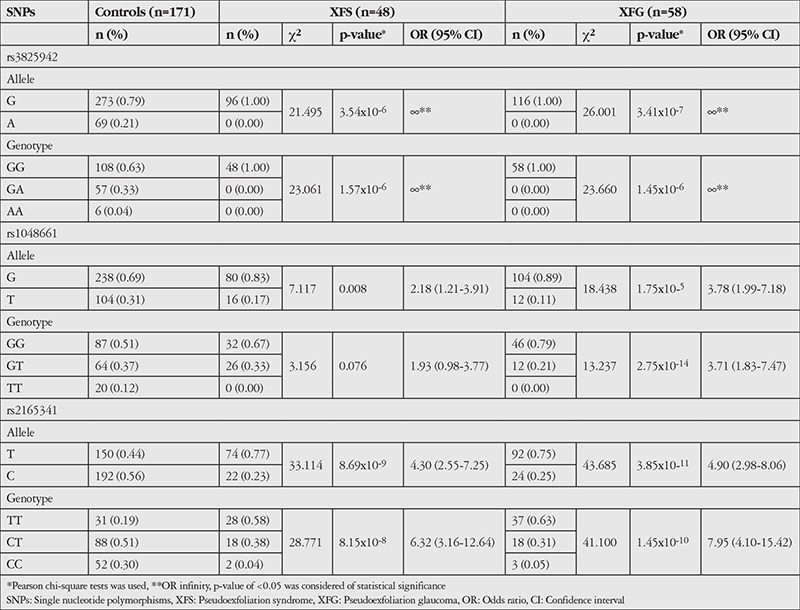
Allele and genotype association analysis for the three single nucleotide polymorphisms of *LOXL1*

**Table 3:**
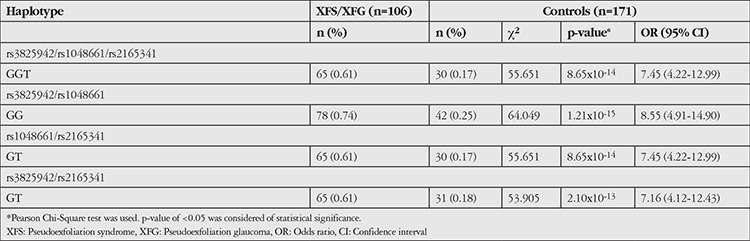
Haplotype analysis of risk alleles at rs3825942, rs1048661 and rs2165341 in combined patients and controls

**Table 4:**
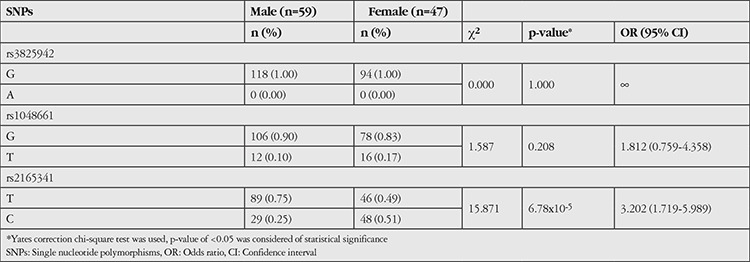
The allele and genotype analysis of the pseudoexfoliation group based on gender
